# Management of Dietary Habits and Diarrhea in Fap Individuals: A Mediterranean Low-Inflammatory Dietary Intervention

**DOI:** 10.3390/nu13113988

**Published:** 2021-11-09

**Authors:** Ciniselli Chiara Maura, Bruno Eleonora, Oliverio Andreina, Baldassari Ivan, Pastori Marta, Signoroni Stefano, Vitellaro Marco, Ricci Maria Teresa, Milione Massimo, Cattaneo Laura, Gariboldi Manuela, Mancini Andrea, Rivoltini Licia, Morelli Daniele, Pasanisi Patrizia, Verderio Paolo

**Affiliations:** 1 Bioinformatics and Biostatistics Unit, Department of Applied Research and Technological Development, Fondazione IRCSS Istituto Nazionale dei Tumori, 20133 Milan, Italy; chiara.ciniselli@istitutotumori.mi.it (C.C.M.); marta.pastori@istitutotumori.mi.it (P.M.); paolo.verderio@istitutotumori.mi.it (V.P.); 2 Unit of Epidemiology and Prevention, Department of Research, Fondazione IRCCS Istituto Nazionale dei Tumori, 20133 Milan, Italy; eleonora.bruno@istitutotumori.mi.it (B.E.); andreina.oliverio@istitutotumori.mi.it (O.A.); ivan.baldassari@istitutotumori.mi.it (B.I.); 3 Unit of Hereditary Digestive Tract Tumors, Department of Surgery, Fondazione IRCCS Istituto Nazionale dei Tumori, 20133 Milan, Italy; stefano.signoroni@istitutotumori.mi.it (S.S.); marco.vitellaro@istitutotumori.mi.it (V.M.); mariateresa.ricci@istitutotumori.mi.it (R.M.T.); 4 First Pathology Division, Department of Diagnostic Pathology and Laboratory, Fondazione IRCCS Istituto Nazionale dei Tumori, 20133 Milan, Italy; massimo.milione@istitutotumori.mi.it (M.M.); laura.cattaneo@istitutotumori.mi.it (C.L.); 5 Unit of Genetic Epidemiology and Pharmacogenomics, Department of Research, Fondazione IRCCS Istituto Nazionale dei Tumori, 20133 Milan, Italy; manuela.gariboldi@istitutotumori.mi.it; 6 Unit of Diagnostic and Therapeutic Endoscopy, Department of Surgery, Fondazione IRCCS Istituto Nazionale Tumori, 20133 Milan, Italy; andrea.mancini@istitutotumori.mi.it; 7 Unit of Immunotherapy of Human Tumors, Department of Research, Fondazione IRCCS Istituto Nazionale dei Tumori, 20133 Milan, Italy; licia.rivoltini@istitutotumori.mi.it; 8 Laboratory Medicine Division, Department of Diagnostic Pathology, Fondazione IRCCS Istituto Nazionale Tumori, 20133 Milan, Italy; daniele.morelli@istitutotumori.mi.it

**Keywords:** FAP individuals, Mediterranean low-inflammatory diet, dietary questionnaires, dietary adherence, diarrheal discharges

## Abstract

Background: A total colectomy and a frequent life-long endoscopic surveillance are guaranteed to patients with Familial Adenomatous Polyposis (FAP) to reduce their risk of duodenal and rectal stump cancers. However, after surgery, individuals with FAP suffer from an increased number of diarrheal discharges that force them to dietary restrictions. A non-randomized pilot study was conducted to assess whether a three-month low-inflammatory Mediterranean dietary intervention reduces gastro-intestinal markers of inflammation in FAP individuals. The aim of the present work is to evaluate the participant’s adherence to the proposed dietary recommendations and the change in their number of diarrheal discharges. Methods: 26 FAP individuals aged >18 years, who underwent a total colectomy with ileo-rectal anastomosis and were involved in the surveillance program at the Fondazione IRCCS Istituto Nazionale Tumori of Milan, were included in the present analysis. Results: FAP individuals significantly reduced the *Not recommended* foods (*p*-value: 0.002) and increased the consumption of the *Recommended* ones (*p*-value: 0.075). The adherence to the proposed dietary recommendations was accompanied by a significant decrease in the number of diarrheal discharges (*p*-value: 0.008). Conclusions: This study suggests that adhering to a low-inflammatory Mediterranean diet has a potential protective effect on the number of diarrheal discharges in FAP individuals.

## 1. Introduction

Familial Adenomatous Polyposis (FAP) is a rare premalignant hereditary autosomal-dominant condition due to germline mutations in the Adenomatous Polyposis Coli (*APC*) gene. FAP is characterized by the development of hundreds to thousands of adenomatous polyps in the colon and progression to cancer in almost 100% of patients if not treated with an early prophylactic surgery [1]. A total colectomy with ileo-rectal anastomosis (TC-IRA) and a frequent life-long endoscopic surveillance are guaranteed to patients to reduce their risk of duodenal and rectal stump cancers [2,3]. However, after surgery, individuals with FAP suffer from many intestinal disorders including increased frequency of defecation, urgency to open bowels, alteration in stool consistency and changes in bowel habits. A recent analysis on people with FAP or Lynch syndrome showed that, despite patients undergoing TC-IRA, they had a similar morbidity and mortality rate compared to those who underwent segmental resection, and they reported a worse bowel function [4].

Intestinal disorders force FAP individuals to dietary restrictions, and they usually employ an elimination diet to identify what they can consume to minimize problems, and, as a result, they usually follow a monotonous diet and plan eating activities differently.

A non-randomized pilot study on FAP individuals [5,6] was conducted to assess whether a three-month low-inflammatory Mediterranean dietary intervention reduces gastro-intestinal markers of inflammation and improves quality of life. The results included significant changes in stool and serum calprotectin levels as markers of local inflammation and borderline changes for the neutrophil-lymphocyte ratio as a marker of systemic inflammation [6].

The aim of the present work is to evaluate, in the FAP individuals enrolled into the pilot study, the adherence to the proposed low-inflammatory dietary recommendations after the three-month intervention. The results of the changes in the number of diarrheal discharges as an important aspect of participants’ quality of life were also reported.

## 2. Materials and Methods

### 2.1. Study Design

Detailed information regarding the study design, protocol and results on inflammation markers was previously reported [5,6]. Briefly, the study was a prospective non-randomized pilot single arm trial, aimed at evaluating the effects of a Mediterranean low-inflammatory diet in terms of the reduction in calprotectin levels in stool. FAP individuals aged >18 years, who underwent TC-IRA and were involved in the surveillance program at the Fondazione IRCCS Istituto Nazionale Tumori of Milan (INT), were eligible. FAP individuals treated with non steroid anti-inflammatory drugs such as COX-2 inhibitors or with omega-3 products were excluded from the trial. The participants signed an informed consent and were asked to donate blood samples (to determinate insulin, insulin-like growth factor-1, C-reactive protein, glucose, calprotectin and glycated hemoglobin) and feces (to determinate fecal calprotectin) at baseline, at the end of the three-month dietary intervention and at six months (respectively, T0, T1 and T2). They were also invited to undergo endoscopy at T0 and T2, in which tissue samples of healthy rectal mucosa and adenomas were collected. Participants’ adherence to the low-inflammatory Mediterranean diet was evaluated through two 24-h food frequency diaries of the previous day’s food intake at T0 and T1.

The study was approved by the Ethical Committee of INT 78/2017 and registered as interventional study #NCT04552405.

### 2.2. Dietary Intervention

The proposed dietary intervention is fully described elsewhere [5,6]. Briefly, the low-inflammatory dietary recommendations were based on Mediterranean diet principles and recipes, and included some fermented food of Japanese tradition (miso, soy sauce, tempeh, humeboshi) to improve bowel function. FAP participants were invited to reduce the consumption of refined food (e.g., sugar, sugary beverages, sweets and refined grains) and of animal fatty food (red/processed meat, butter, fatty cheese) and were encouraged to improve their dietary quality by increasing plant and unrefined foods. The diet also included the use of prebiotics and probiotics (fresh cultured yogurt, kefir, Manuka honey) to restore the balance of the intestinal flora and a modification of the textures of foods (e.g., blending, grinding or cooking) as needed depending on the patient’s symptoms.

The dietary intervention included 12 days of nutritional activities including cookery classes, supportive educational content and common meals.

### 2.3. Dietary Questionnaires

The 24-h food frequency diaries of this pilot study were already used and described in previous dietary intervention trials [5,7,8].

The 24-h food frequency diary contains a list of 65 food items, without any information on portion size or weight, or on recipes. Individuals only had to indicate whether, on the previous day, they had eaten or not eaten the specified food at breakfast, lunch, dinner and breaks. The diary is organized in the following six groups:

1. **Drinks** (including alcohol), **milk and dairy products**. This group includes also plant-based unsweetened beverages.
2. **Sweets and confectionery** including sugar, whole sugar, honey and malt.
3. **Bread and grains**. This group has items for refined grains, whole grains and grain products.
4. **Meat, fish, eggs and meat substitutes** that include red and processed meat, white meat and meat substitutes made from wheat gluten protein.
5. Legumes, vegetables, fresh and dried fruit, nuts, seeds and soy products.
6. **Sauces, animal and vegetable fats,** including also fermented soy condiments (miso and soy sauce).

The short questionnaire on the adherence to the Mediterranean diet (MEDAS) [9] was previously described [5]. Briefly, it consists of twelve questions on food consumption frequency and two on eating habits. Each question is scored 0 or 1 depending on whether participants adhere to each Mediterranean component or not. The total MEDAS score is given by the sum of the single scores and ranges from 0 to 14. FAP participants completed the 24-h food frequency diaries and the MEDAS questionnaires at baseline, before starting the dietary intervention (T0) and at the end of the three-month dietary intervention (T1).

### 2.4. Food Categorization

Data from the 24 h food frequency diaries about the previous day’s consumption were reported as single food and food groups. Groups of recommended, not recommended or neutral foods were created on the basis of the proposed low-inflammatory dietary recommendations (Supplementary Materials Table S1).

The group of *Recommended* foods included:

− Vegetable milk, tea, barley coffee, unsweetened yogurt, brown sugar, honey, malt, unsweetened jam, durum wheat bread and pasta, durum wheat pizza, unsweetened muesli, oat flakes, fish, legumes, tofu/tempeh, onions, tomato juice, tomato sauces, green and black olives, cooked vegetables (zucchini, pumpkin etc.), row vegetables, berries, pomegranate, apples, pears, unsweetened fruit juices, other kind of fresh fruits, dried fruits, dried oleaginous fruits (including in unsweetened creams), extra-virgin olive oil, miso, tamari, mushrooms.

The group of *Not recommended* foods included:

− Animal milk, dairy products, “ricotta”, sugary beverages, alcoholic drinks (wine, beer, spirits), white sugar, artificial sweeteners, sweetened jam, milk chocolate, candies, biscuits, ice creams, brioches, egg noodles, corn flakes, sweetened muesli, red meat, processed meat, potatoes, mashed potatoes, French fries, butter, lard cream, margarine, ready sauces, mayonnaise, ketchup.

*Neutral* foods (not recommended but not discouraged) included:

− -Café, unsweetened fruit juices, dark chocolate, cookies, ice cream or other desserts without sugar, white bread (and pizza), whole meal flour bread and pizza, white rice, whole rice, rice cakes, other grains, white meat, clams and shellfish, seitan, eggs, artichokes and fennel, cabbage family, peppers and eggplants, citrus fruit, fresh unsweetened fruit juices, seed oil, spices.

Groups of *Recommended* or *Not recommended* foods were also created using data from MEDAS. According to the MEDAS items, vegetables, fruits, legumes, fish and nuts were included in the *Recommended* foods while sugary beverages, sweets, red/processed meat and animal seasonings entered in the *Not recommended*.

### 2.5. Statistical Analysis

Standard descriptive statistics—medians and IQR for continuous variables and frequency tables for categorical variables—were used to describe the study sample and the pivotal variable distributions. Then, for each of the above-mentioned food categories (*Recommended, Not Recommended* and *Neutral)*, an overall score was computed by summing-up the intake frequency (score_R, score_NR, score_N) at baseline (T0) and 3-month (T1), respectively. These scores were the pivotal variables used for the subsequent statistical analysis. The nonparametric Wilcoxon signed-rank test (WSRT) was used to compare the baseline and 3-month distributions of the food intake scores as well as the number of diarrheal discharges [10]. To evaluate the changes between T0 and T1, a five-scale classification criterion was adopted by jointly considering the score intakes of *Recommended* and *Not Recommended* food at T0 and T1 as reported in Supplementary Materials Table S2. Stacked bar charts were used to depict the pattern of each of the 65 items of the 24-h food frequency diary data at baseline (T0) and after the three months of intervention (T1). A similar approach was also used to highlight changes in the food frequency intake of *Recommended* and *Not recommended* foods using data from MEDAS. All statistical analyses were carried out with SAS software (Version 9.4; SAS Institute Inc, Cary, NC), adopting a significance level of α = 0.05.

## 3. Results

Out of 34 FAP individuals enrolled, six patients dropped out before starting the dietary intervention, and two others, despite participating in the entire dietary intervention, did not complete the MEDAS (one did not complete the first, the other the final one). Therefore, 26 subjects, 13 men and 13 women with a median age at baseline of 43 years (range 18–77), were included in the present analysis.

### 3.1. Food Intake Distributions

**Figure 1:**
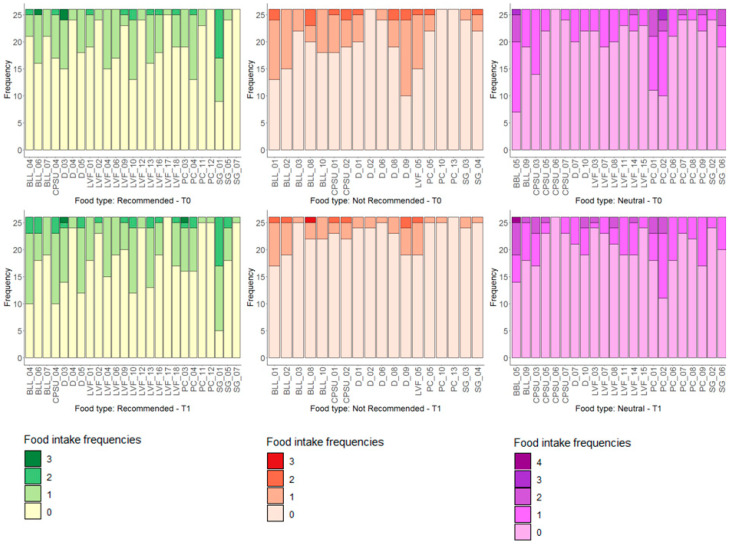
Distribution of food intake of Recommended, Not Recommended and Neutral at T0 (upper part) and T1 (bottom part). On the x-axis are reported the dietary items considered and on the y-axis the number of subjects according to the absolute frequency of intake.

Figure 1 depicts the pattern of the 24-h food frequency diaries on the previous day’s consumption in term of the number of portions as single foods stratified according to the *Recommended, Not Recommended* and *Neutral* groups at T0 and T1.

By looking at the overall scores (score_R, score_NR, score_N), computed by summing-up the intake frequencies, Table 1 reports the main descriptive statistics together with the results of the Wilcoxon signed-rank test.

**Figure 2:**
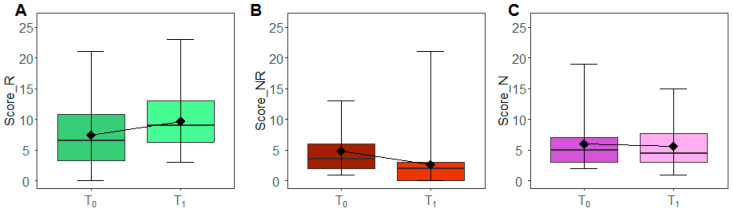
Scores modulation. The box plots report the overall distribution of Recommended (**A**), Not Recommended (**B**) and Neutral scores (**C**) at T0 and T1. Each box indicates the 25th and 75th centiles. The horizontal line inside the box indicates the median; the black diamond represents the mean; the whiskers indicate the range measure.

Boxplots in Figure 2 depict the pattern of the overall scores at baseline (T0) and after the three months of intervention (T1). A statistically significant decrease (WSRT *p*-value: 0.002) of *Not Recommended* foods was observed, with a median frequency of 3.5 and 2 at T0 and T1, respectively. Consistently, we observed an increment of *Recommended* foods between T0 and T1 (median T0: 6.5, median T1: 9) even if with a borderline significance (WSRT *p*-value: 0.075) and a substantial stability of the *Neutral* food ones (WSRT *p*-value: 0.977). By looking at the overall changes between T0 and T1 (Supplementary Materials Figure S1), 53.85% of patients decremented the intake of *Not Recommended* foods or maintained their intake by increasing or maintaining in parallel the intake of *Recommended* foods. On the contrary, none of the patients incremented the intake of *Not Recommended* foods or decreased those of the *Recommended* ones. Moreover, 30.77% of the subjects decremented the intake of *Recommended* foods with a decrement or stability of *Not Recommended* foods.

### 3.2. Diarrheal Discharges

**Figure 3:**
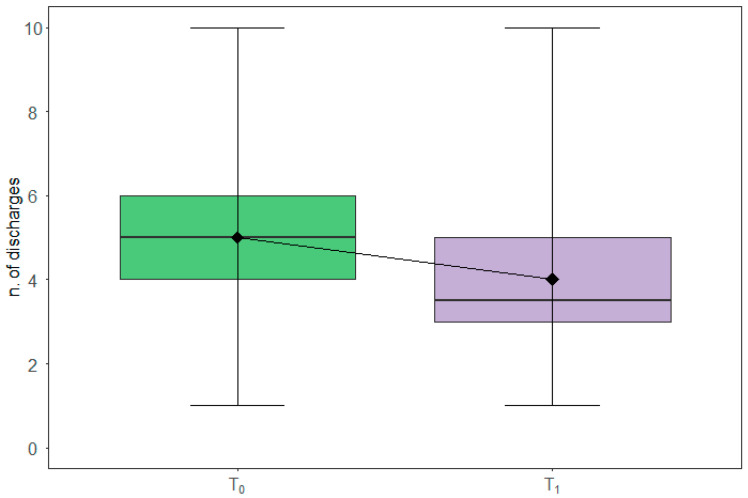
Diarrheal discharges modulation. The box plots report the overall distribution of diarrheal discharges at T0 and T1. Each box indicates the 25th and 75th centiles. The horizontal line inside the box indicates the median; the black diamond represents the mean; the whiskers indicate the range measure.

Boxplots in Figure 3 depict the corresponding pattern at baseline (T0) and after the three months of intervention (T1) as assessed by the 24-h food frequency diaries, with a significant reduction of an average of one diarrheal discharge (WSRT *p*-value: 0.008) between T0 and T1.

### 3.3. Diarrheal Discharges and Food Intake

**Figure 4:**
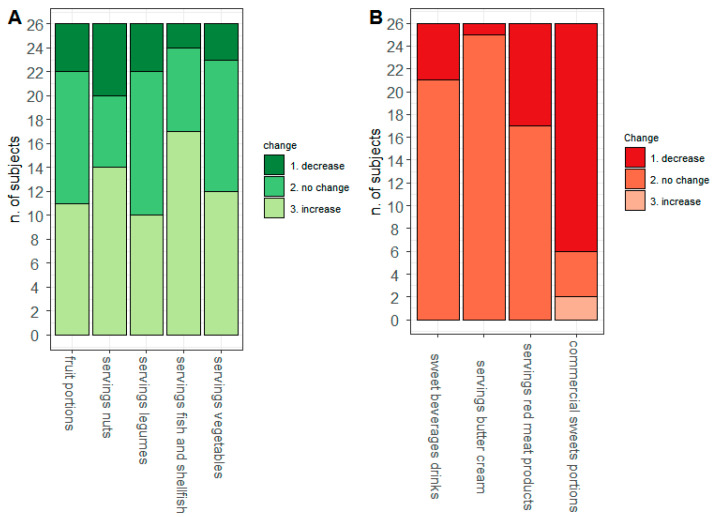
MEDAS modulation. On the x-axis are reported the dietary items considered and on the y-axis the number of subjects according to the absolute frequency of Recommended (**A**) and Not Recommended (**B**) intake.

By looking at the number of diarrheal discharges as assessed by the MEDAS questionnaire, Figure 4 depicts the change between T0 and T1 of the food intake of the selected items. Interestingly, we found that FAP patients increased the intake of some recommended foods (Panel A)—especially fish and nuts—whereas they reduced the intake of commercial sweets (Panel B).

**Figure 5:**
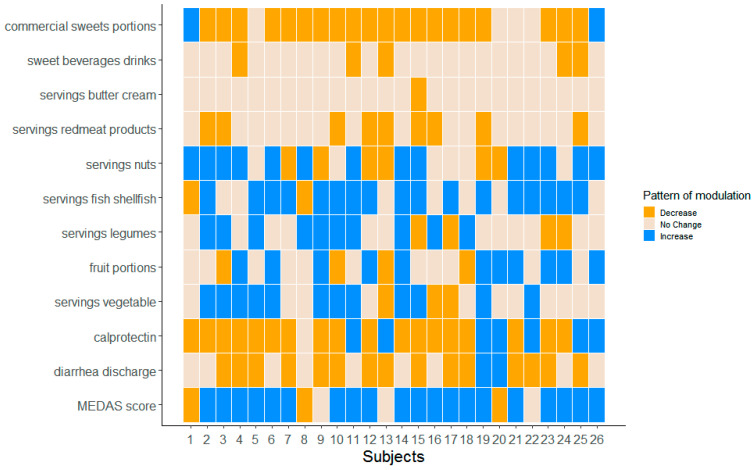
Heatmap FAP-patient level. Representation of the MEDAS questionnaire data of the 26 considered patients together with the number of diarrheal discharges and fecal calprotectin.

Finally, we pursue the analysis by looking at the relationships between the number of diarrheal discharges and the MEDAS score as well as the fecal calprotectin, which was found to be decreased at T1 compared to T0 [6]. Figure 5 represents the subject-level heatmap showing the modulation in the overall MEDAS score, diarrheal discharges, fecal calprotectin (on the bottom) and the food intakes arising from the MEDAS questionnaire. We observed an encouraging reduction in diarrheal discharges and fecal calprotectin accompanied by an increase in the MEDAS score. Moreover, patients reporting lower diarrheal discharges after the three-month dietary intervention improved the intake of Recommended foods, especially fish/shellfish, vegetables and legumes, and reduced the intake of Not Recommended, especially commercial sweets.

## 4. Discussion

Due to the chronic condition and the intensive program of endoscopic surveillance, FAP individuals attend healthcare services along their lives. Following prophylactic colectomy and during outpatient check-ups, FAP patients systematically ask for a dietary support for the management of too many discharges, sometimes for a bloated stomach, sometimes for fear that some foods can worsen their symptoms and stimulate the formation of new adenomas. Our previous results showed that a Mediterranean low-inflammatory diet reduces markers of local intestinal inflammation (stool and serum calprotectin) and the neutrophil-lymphocytes ratio as an index of systemic immune inflammation [5] as well as significantly improving the overall MEDAS scores [6].

The present work shows results on the adherence of the proposed dietary intervention using two different tools, the 24-h food frequency diaries and the MEDAS questionnaire in order to collect different and complementary kinds of information. Diaries registered food consumption in the previous day. Taking sweets as an example, people substantially had to answer to the question “Did you consume sweets yesterday?”, and, in the case of a positive answer, they had to indicate how many times in that day. On the other hand, the MEDAS registered data of food consumption on average (per day or per week). People substantially had to answer to the question “How many servings of sweets do you usually eat per week?”. Our dietary results are consistent by using data from both diaries and the MEDAS.

We aimed to achieve in FAP individuals a healthier dietary pattern, mainly based on plant and unrefined foods. Patients were encouraged to avoid sugary beverages/foods, to reduce red/processed meat and to privilege the consumption of foods rich in omega-3 such as fish (salmon, mackerel, anchovies, sardines), nuts and seeds (especially in creams without sugar for the preparation of desserts). Nuts and legume flours were proposed in sweet and savory cookery recipes. Along the three-month dietary intervention, the FAP individuals included in our pilot study adhered to the proposed low-inflammatory dietary recommendations, by significantly reducing the Not recommended foods (especially sweets) and by increasing the consumption of the Recommended ones (especially fish). This improvement in participants’ dietary habits was accompanied by a significant decrease in the number of diarrheal discharges, and data suggested a trend of a negative association between the number of diarrheal discharges and both the adherence to the proposed recommendations and fecal calprotectin. Interestingly, taking a look at the heatmap, participants who did not reduce or increased the number of diarrheal discharges were mainly those who did not modify the consumption of Not Recommended foods and partially increased the Recommended ones. The group of *Recommended* foods includes some core constituents of the Mediterranean diet such as vegetables and legumes which, due to the higher contents in fibers, were difficult to manage by FAP participants, although cooked and passed through a sieve. In a recent study, healthy people with a higher adherence to the Mediterranean diet showed a beneficial gut microbiota but also experienced a higher defecation frequency and a mild gastrointestinal symptomatology [11]. Dietary fiber intake, fermentation and a bulking-effect caused directly via water retention were significant contributors in these associations [12]. These considerations and the results reported in the subject-level heatmap suggest, for a further future dietary intervention in FAP individuals, to start at first by reducing pro-inflammatory foods (red/processed meat) and certain carbohydrates that cause dysbiosis (lactose and in general refined sugars), and then incrementing the low-inflammatory ones, mainly those higher in fiber content.

Few studies reported on bowel function and quality of life in patients after TC-IRA by using different questionnaires such as SF-36, EORTC QLQ-C38 or COREFO [4,13,14,15,16]. A limitation of our study is the lack of a specific validated questionnaire to assess bowel function. An additional single question about the number of diarrheal discharges was included both in the 24-h diary and in the MEDAS questionnaire. The generalizability of these results is therefore limited. However, we were interested in recording a simple clinical sign of bowel function in response to the diet, and a change in the number of diarrheal discharges emerged from both the previous 24-h diary and the MEDAS.

Bowel function may be modulated via consumption of probiotics—live microbes which interacting with gastrointestinal microbiota and consumed at a sufficient dose provide beneficial effects [17]. The easiest and most common way to introduce potentially beneficial microbes is via consumption of microbe-containing foods, and fermented foods and beverages, in particular. FAP participants were strongly encouraged to consume fresh cultured yogurt, Kefir and fermented soy products (especially miso) every day. Fermentable fibers and probiotics are further dietary components known to improve bowel function. In human inflammatory bowel diseases, the consumption of inulin-type fructans was shown to reduce fecal calprotectin and increase *Bifidobacteria* in the luminal microbiota [18,19]. However, especially along the active phases of inflammatory bowel diseases, fermentable carbohydrates have been associated with exacerbation of functional symptoms. Therefore, although there are no data on individuals with FAP, participants in our trial were recommended to consume onions, garlic, chicory, leeks, oats, barley (containing betaglucans, oligofructose and inulin) only in the last phases of the intervention, and depending on their bowel symptoms.

A further limitation of our study is that we are unable to identify which food group or food was relevant for the assessed results. We certainly proposed a comprehensive change in diet that, reducing pro-inflammatory substances and improving bioactive protective compounds, might explain the effects on inflammation and diarrheal discharges.

Chemoprevention to block or delay the process of carcinogenesis has attracted interest in patients at high risk of developing cancer [20]. Although non-steroidal anti-inflammatory drugs are able to reduce the number of polyps in FAP, severe cardiovascular effects have been reported [20], pointing out the need to find alternative treatments without the risk of developing side effects. Experimental models recently suggested that dietary supplements (phytoestrogens, insoluble fibers, probiotics, quercetin, *n*-3 polyunsaturated fatty acids) might be useful to reduce intestinal carcinogenesis in rats mutated in the APC gene [21,22,23,24]. Recently, a small non-randomized study of three-month oral supplementation with phytoestrogens and indigestible insoluble fibers showed a reduction in the number and size of duodenal polyps in FAP individuals [25].

Our intervention study suggests that adhering to a low-inflammatory Mediterranean diet has a potential protective effect on markers of inflammation and the number of diarrheal discharges in FAP individuals. The strengths of this study are its novelty and feasibility. These encouraging results open new perspective for the clinical management of FAP disease and represent the basis for larger randomized trials to evaluate the effects of the low-inflammatory diet on adenoma recurrence in these patients.

## Figures and Tables

**Table 1:** Descriptive statistics of the three overall scores.

Variables	*n*	min	25th Centile	Median	75th Centile	Max	IQR	*p*-Value *
Recommended (score_R)								
T0	26	0	3	6.5	11	21	8	0.075
T1	26	3	6	9	13	22	7	
Not Recommended (score_NR)								
T0	26	1	2	3.5	6	13	4	0.002
T1	26	0	0	2	3	21	3	
Neutral (score_N)								
T0	26	2	3	5	7	19	4	0.977
T1	26	1	3	5	8	16	5	

* *p*-value of the Wilcoxon signed-rank test.

## Data Availability

Data are available on request.

## Supplementary Materials

The following are available online at https://www.mdpi.com/article/10.3390/nu13113988/s1, Table S1: Groups of *Recommended*, *Not Recommended* or *Neutral* foods; Table S2: Five-scale classification criteria of *Recommended* and *Not Recommended* foods at T0 and T1; Figure S1: Distribution of the food intake changes between T0 and T1.
