# Supplementary material for: Management of Dietary Habits and Diarrhea in Fap Individuals: A Mediterranean Low-Inflammatory Dietary Intervention
Source: Nutrients. 2021 Nov 9;13(11):3988. doi: 10.3390/nu13113988 (PMC8623170; doi:10.3390/nu13113988)
Supplement: Supplementary file 1 [file nutrients-13-03988-s001.zip › nutrients-1443448-supplementary.pdf]

Supplementary Material

Table S1. Groups of *Recommended, Not Recommended* or *Neutral* foods

**Label**	**Food decoding**
Food: *Recommended*	
BLL_04	Vegetable milk without sugar (rise, oats, soybeans, almonds)
BLL_06	Tea, herbal tea, barley
BLL_07	Unsweetened natural yogurt
D_03	Brown sugar, honey, maple syrup, agave syrup
D_04	Malts
D_05	Jam without sugar
PC_03	Bread and pizza made with durum wheat flour
PC_04	Pasta (like spaghetti or macaroni)
PC_11	Oats, oatmeal
PC_12	Muesli without sugar
CPSU_04	Fish
LVF_01	Legumes (beans, lentils, chickpeas, beans, green/red soybeans etc.)
LVF_02	Yellow soybeans, tofu, tempeh
LVF_04	Onions, leeks, garlic
LVF_06	Tomato, tomato juice, tomato sauces
LVF_09	Green and black olives
LVF_10	Other cooked vegetables (zucchini, pumking etc.), vegetable soups
LVF_11	Other raw vegetables (lettuce, other kind of salad, cucumbers etc.)
LVF_12	Berries, pomegranate (including unsweetened fresh juices)
LVF_13	Apples, pears
LVF_16	Other kind of fresh fruits
LVF_17	Dried fruits, raisins, prunes, apricots
LVF_18	Walnuts, hazelnuts, almonds (including creams), oiled seeds
SG_01	Extra-virgin olive oil
SG_05	Miso, Tamari, tekka
SG_07	Mushroom
Food: *Not Recommended*	
BLL_01 _01	Cow or goat's milk, any kind
BLL_02	Fresh/seasoned cheese of any kind (including those that are used for pizza or pasta)
BLL_03	Ricotta, with/without salt
BLL_08	Coca-Cola or other sweetened commercial beverages<br>(fruit juices, vegetable milks and yogurt)
BLL_10	Alcoholic drinks (wine, beer, spirits etc. etc.)
D_01	Refined white sugar
D_02	Artificial sweeteners
D_06	Jam with sugar or fructose, glucose, dextrose
D_08	White or milk chocolate, candies

D_09	Cookies, brioches, sweets, brand ice cream
PC_05	Handmade egg pasta, pasta made with soft-wheat flour
PC_10	Corn, corn flakes
PC_13	Muesli with honey or sugar
CPSU_01	Red meet (beef and veal, sheep, porks, horses)
CPSU_02	Processed meat (salami, ham, sausages, canned meat)
LVF_05	Potatoes, potato chips, mashed potatoes, dumplings
SG_03	Butter, cream, suet, lard, margarine
SG_04	Souces, nuts, mayonnaise, ketchup etc.
Food: *Neutral*	
BLL_05	Cafè
BLL_09	Unsweetened fruit juices
D_07	Dark chocolate
D_10	Cookies, ice cream or other desserts without sugar, fructose, glucose and hydrogenated fats
PC_01	White bread, crackers, biscuits, pretzels, bread sticks, pizza
PC_02	Bread, pizza or crisp bread made with wholemeal flour or partial wholemeal flour
PC_06	White rice, puffed rice
PC_07	Whole rice/partial whole rice, basmati rice
PC_08	Rise cakes
PC_09	Spelt, barley, millet, buckwheat (grains or flakes), quinoa
CPSU_03	White meat or game (chicken, rabbit, turkey, ostrich, pheasant, etc. etc.)
CPSU_05	Clams and shellfish
CPSU_06	Seitan
CPSU_07	Eggs, omelette
LVF_03	Artichokes, cardoons, fennel
LVF_07	Peppers, eggplants
LVF_08	Cabbage, cauliflower, broccoli, turnips, radishes, arugula, daikon, beetroots
LVF_14	Citrus
LVF_15	Fresh unsweetened fruit juices
SG_02	Seed oil
SG_06	Spices

Table S2. Five-scale classification criteria of *Recommended* and *Not Recommended* foods at T0 and T1

Recommended food intake (Δ_R = T1 – T0)	*Not Recommended* food intake (Δ_{NR} = T1 – T0)	category	description
$\Delta_R = 0$	$\Delta_{NR} < 0$	1	Stability or decrease of *NR* foods and stability or increase of *R* foods
$\Delta_R > 0$	$\Delta_{NR} = 0$		
$\Delta_R > 0$	$\Delta_{NR} < 0$		
$\Delta_R = 0$	$\Delta_{NR} = 0$	2	No changes
$\Delta_R = 0$	$\Delta_{NR} > 0$	3	Increase of *NR* foods and stability or increase of *R* foods
$\Delta_R > 0$	$\Delta_{NR} > 0$		
$\Delta_R < 0$	$\Delta_{NR} = 0$	4	Decrease of *R* foods and stability or decrease of NR foods
$\Delta_R < 0$	$\Delta_{NR} < 0$		
$\Delta_R < 0$	$\Delta_{NR} > 0$	5	Decrease of *R* foods and increase of *NR* foods

Figure S1. Distribution of the food intake changes between T0 and T1.

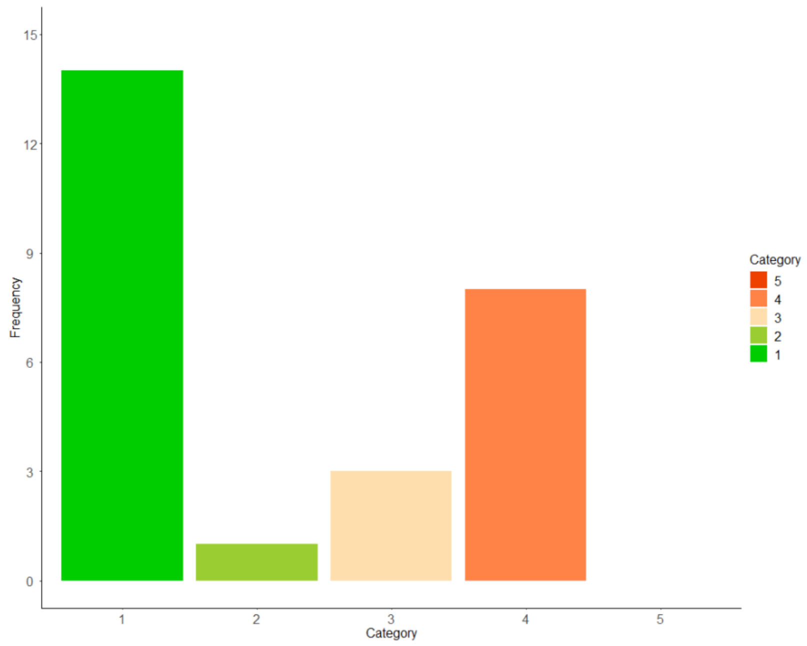

Figure S1. *Bar plot reporting the changes in the food intake frequency as aassed by the diaries by jontly considering the intake modulation of Recommented and Not Recommended foods.*
